# Optimization of Ovum Pick-Up and Embryo Transfer Techniques for Enhancing Reproductive Efficiency in Nili-Ravi Buffaloes

**DOI:** 10.3390/ani16132086

**Published:** 2026-07-06

**Authors:** Yangyang Fan, Bo Wang, Leyi Wang, Cuijin Teng, Jinping Xiong, Jiaqi Jiang, Qingyou Liu, Kuiqing Cui, Zhipeng Li

**Affiliations:** 1 Guangxi Key Laboratory of Animal Reproduction, Breeding and Disease Control, College of Animal Science and Technology, Guangxi University, Nanning 530004, China; yyfan2026@126.com (Y.F.); 18892893218@163.com (B.W.); 2 Royal Cell Biological Technology (GUANGXI) Co., Ltd., Nanning 530009, China; wangleyi959@163.com (L.W.); tcjcpa@163.com (C.T.); piaoxuely@163.com (J.X.); 3 Guangxi Zhuang Autonomous Region Buffalo Milk Quality and Safety Control Technology Engineering Research Center, Guangxi Buffalo Research Institute, Chinese Academy of Agricultural Sciences, Nanning 530001, China; jjqi2024@163.com; 4 College of Animal Science, South China Agricultural University, Guangzhou 510642, China; qingyouliu@scau.edu.cn; 5 School of Animal Science and Technology, Foshan University, Foshan 528225, China

**Keywords:** Nili-Ravi buffalo, ovum pick-up (OPU), embryo transfer (ET), estrus synchronization, reproductive efficiency

## Abstract

Nili-Ravi buffalo is a core dairy breed for China’s genetic improvement, yet its assisted reproductive technologies are restricted by low ovum pick-up (OPU) efficiency and unstable embryo transfer (ET) pregnant rates. This study established statistically optimized key parameters for OPU procedures in Nili-Ravi buffalo, and evaluated the effects of multiple ET-related protocols. For OPU, 50–55 mmHg vacuum pressure balanced oocyte recovery and usable rate best (*p* < 0.05). Young and adult buffalo performed significantly better than prepubertal heifers (*p* < 0.05). Four consecutive weekly consecutive OPU sessions did not significantly reduce oocyte yield (*p* > 0.05). FSH treatment markedly enhanced oocyte quantity, quality and recovery efficiency (*p* < 0.05), with no significant difference between two FSH protocols (*p* > 0.05). For ET, three estrus synchronization protocols showed no significant effects on estrus, ovulation, transferable recipient and pregnant rates in breeding or non-breeding seasons (*p* > 0.05), though optimized protocols showed a numerical improvement. Combinations of CL grade and embryo quality, as well as post-transfer treatments with vitamin ADE, hCG and progesterone, did not significantly affect pregnancy or early abortion rates (*p* > 0.05). This study built a practical reference framework for OPU-ET procedures in Nili-Ravi buffalo, with key OPU parameters statistically optimized and ET protocols showing numerical improvements that require further validation with larger sample sizes.

## Simple Summary

Nili-Ravi buffalo are key dairy animals in China, but getting them to reproduce efficiently using modern assisted reproductive techniques—such as collecting egg cells from live donor buffalo and transferring embryos into recipient buffalo—has been difficult due to low egg collection success and unstable pregnant rates. This study optimized key steps of the procedure, including suction pressure for egg collection, donor age, repeated egg collection, hormone treatments, and embryo transfer care. We found that a mild suction pressure, using young or adult donors, and weekly collection for four consecutive weeks yielded stable oocyte output, and FSH treatment significantly improved both the quantity and quality of recovered oocytes. For embryo transfer, adjusted synchronization and post-transfer care showed helpful trends, though results were not statistically significant. Overall, this study provides a practical, optimized technical reference for OPU and ET operations in Nili-Ravi buffalo, which can support the efficient expansion of high-quality dairy buffalo germplasm and promote the sustainable development of the dairy buffalo industry.

## 1. Introduction

Buffalo is a pivotal dairy livestock species in tropical and subtropical regions. China is one of the major countries for buffalo breeding, but most stocks are low-productivity swamp-type local breeds. As superior river-type dairy breeds, Murrah and Nili-Ravi buffalo are characterized by high milk yield, elevated milk fat and protein content, strong roughage adaptability, and high disease resistance [1,2], thus serving as the core parental germplasm for dairy buffalo genetic improvement in China [3]. Nili-Ravi buffalo, as a superior river-type dairy breed, has an average annual milk yield of 2000–3000 kg with 7–8% milk fat content, which is 3–4 times higher than the local swamp-type buffaloes (500–800 kg/year, 5–6% milk fat), making it the core parental germplasm for dairy buffalo genetic improvement in China. However, traditional crossbreeding requires multigenerational selection with a breeding cycle exceeding 12 years, failing to rapidly satisfy the demand for high-performance breeding stock. Meanwhile, long-term closed breeding has led to severe inbreeding depression in Chinese buffalo populations, resulting in continuous deterioration of production performance, with milk yield notably lower than that of the original breeds. Insufficient efficiency of elite germplasm expansion has become a critical bottleneck restricting the industrial upgrading of the dairy buffalo sector. Furthermore, buffalo are seasonal breeding animals with indistinct estrus symptoms, unpredictable ovulation timing, prolonged calving intervals, and low natural pregnant rates [4]. Conventional reproductive management models are incompatible with large-scale intensive breeding. Therefore, developing efficient assisted reproductive technologies is urgently required to overcome these constraints.

The integrated technical system of ovum pick-up (OPU), in vitro embryo production (IVP), estrus synchronization (ES), and embryo transfer (ET) constitutes the core platform for genetic improvement and elite germplasm amplification in modern livestock breeding [5,6]. In dairy cattle, OPU-IVP-ET has achieved large-scale commercial application [7]. After technical optimization, Holstein cattle can yield an average of 14 usable oocytes per OPU session and 4.6 transferable embryos per donor, with ET pregnant rates stably exceeding 44% [8,9]. B-ultrasound-guided OPU has become the dominant approach for in vivo oocyte retrieval in cattle due to clear imaging, high oocyte recovery rate, and minimal ovarian trauma [6]. Combined with FSH-induced superstimulatory treatment, OPU can significantly increase the number of recovered oocytes and the proportion of high-quality oocytes [8,9]. In addition to hormonal treatments, nutritional supplementation with vitamins A, D, and E has been shown to improve reproductive outcomes in ruminants by maintaining reproductive epithelial integrity, enhancing antioxidant capacity, and regulating immune function [10,11]. However, the efficacy of vitamin supplementation in buffalo ET programs remains understudied, warranting further investigation. In buffalo reproduction, researchers have established the OPU-IVP-ET system and successfully promoted buffalo breeding [12]. Previous studies pioneered the production of purebred river-type test-tube buffalo using local swamp-type buffalo as recipients, shortening the breed improvement cycle in China [13]. Estrus synchronization protocols such as CIDR-PG and GnRH-PG-GnRH enable centralized estrus expression in recipients and markedly improve the efficiency of large-scale ET [14,15]. Despite the progressive maturation of the technical system, challenges including low OPU efficiency (typically 3–6 usable oocytes per session in local buffalo breeds) and unstable ET pregnant rates (20–30% for frozen-thawed embryos) still limit the large-scale popularization and application of these technologies in buffalo [16,17].

Currently, several critical issues remain unresolved in assisted reproductive technologies for Chinese dairy buffalo. Specifically, the regulatory mechanisms of equipment parameters, donor physiological status, and hormonal treatments on OPU efficiency remain unclear, leading to large fluctuations in oocyte yield and quality. Moreover, the effects of estrus synchronization regimens, luteal developmental status, and post-transfer pregnancy maintenance treatments on ET pregnant rate have not been systematically elucidated, restricting the stability and efficiency of the elite germplasm propagation system. To address these bottlenecks, we hypothesized that OPU efficiency and ET outcome in Nili-Ravi buffalo can be improved by optimizing aspiration pressure, donor status, FSH stimulation, recipient synchronization, and post-transfer support. Therefore, this study systematically investigated the effects of key factors on OPU efficiency and ET pregnant rate in Nili-Ravi dairy buffalo. Using Nili-Ravi buffalo as oocyte donors and Murrah-Nili-Ravi crossbred buffalo as ET recipients, this study optimized core procedures of OPU and ET, and systematically evaluated the effects of key technical parameters on OPU efficiency and ET pregnant rate, and screened a practical parameter combination for field application. The findings will provide a practical reference framework for efficient IVP and ET in dairy buffalo, effectively enhance the efficiency of elite germplasm amplification, and possess important theoretical value and application prospects for alleviating the shortage of superior breeding stock and promoting the sustainable and high-quality development of the dairy buffalo industry.

## 2. Materials and Methods

### 2.1. Materials

Unless otherwise stated, all chemical reagents and culture media were purchased from Sigma-Aldrich Corp. (St. Louis, MO, USA). Disposable plasticware was obtained from Thermo Fisher Scientific Inc. (Shanghai, China). Fetal bovine serum (FBS) was purchased from Gibco Life Technologies (Gaithersburg, MD, USA), and Research Vitro Cleave Medium (K-RVCL) was acquired from William A. Cook (Brisbane, Australia). The Veterinary B-ultrasound EVO Live-Ovary Sampling Instrument and vacuum pump used in this study was purchased from Boxianglai Electronic Sci-tech Co., Ltd. (Zhengzhou, China). The FSH, GnRH, PGF2α, and hCG used in this study were purchased from Sansheng Biological Technology company (Ningbo, China). The Vitamin ADE, penicillin, and streptomycin were purchased from Hebei Yuanzheng Pharmaceutical Co., Ltd. (Shijiazhuang, China).

### 2.2. Experimental Animals and Ethical Approval

Ovum pick-up and in vitro embryo production were conducted at Royal’s JW Buffalo Disease-Free Farm, Punjab, Pakistan. Embryo transfer was performed at a commercial farm affiliated with Royal’s Cell Co., Ltd., Nanning, China. Oocytes were collected in Pakistan, matured, fertilized, and cultured to the blastocyst stage in vitro. Blastocysts were vitrified as described in Section 2.9 and transported to China via international air freight in liquid nitrogen dry shippers, maintaining a temperature below −150 °C throughout the journey. All transport and import procedures complied with the animal health and quarantine regulations of both Pakistan and China, with official veterinary health certificates issued by the relevant authorities. Upon arrival, embryos were stored in liquid nitrogen until transfer at the farm in Nanning, China. All experimental animals were clinically healthy female buffalo with normal reproductive function, complete pedigree records, and no hereditary defects. All OPU donors were purebred Nili-Ravi buffaloes during non-lactating and at least 6 months post-calving (for parous animals). For age groups, prepubertal heifers (12–17 months, 0 parities), young buffaloes (24–30 months, 0–1 parities), adult buffaloes (36–60 months, 1–3 parities). The body weight was 450–700 kg (prepubertal: 450–500 kg; young: 500–600 kg; adult: 550–700 kg). ET recipients were F1 crossbred buffalo (Murrah × Nili-Ravi), aged 3–5 years with 0–2 parities, body weight 400–600 kg, and regular estrous cycles. All recipients had completed routine immunization in accordance with farm management protocols and were housed in indoor pens with ad libitum access to feed and water. All experimental procedures were approved by the Animal Care and Use Committee of Guangxi University (Approval Number: GXU-2024094) and complied with international guidelines for the ethical use of experimental animals.

### 2.3. Conventional OPU Procedure

Ovum pick-up was performed by skilled technicians in a dedicated oocyte collection room with ambient temperature maintained at 20–25 °C using a veterinary B-ultrasound-guided OPU system (Boxianglai Electronic Sci-tech Co., Zhengzhou, China). After restraint in a holding chute, each donor buffalo received epidural anesthesia with 3–5 mL of 5% procaine hydrochloride at the lumbosacral junction. Following the onset of anesthesia, rectal feces were removed, and the vulva was disinfected with 0.1% bromogeramine and dried. Ovarian morphology and follicular population were evaluated via transrectal palpation combined with B-mode ultrasonography. During the procedure, the target follicle was aligned with the puncture guide line and punctured at the moment of clearest imaging and minimal distance from the needle tip. Follicular fluid was aspirated using an automatic pressure-controlled pump until complete collapse of the follicular structure was observed ultrasonographically. Trans-ovarian puncture was avoided to minimize ovarian hemorrhage and tissue damage, and adjacent follicles were aspirated consecutively to reduce repeated punctures. Following oocyte retrieval, the collection tubing was flushed with phosphate-buffered saline (PBS) to remove residual blood and tissue debris. Collection data were recorded in real time, and follicular fluid was transferred to the embryo laboratory through an aseptic window for oocyte recovery and morphological evaluation.

### 2.4. Experimental Design for OPU Optimization

To clarify the effects of key operational parameters on OPU efficiency, four experiments were conducted to optimize aspiration pressure, donor age, OPU frequency, and FSH superstimulatory treatment.

For aspiration pressure optimization, 30 healthy adult buffalo were randomly allocated to three groups (*n* = 10 per group) with aspiration pressures set at 45–50 mmHg, 50–55 mmHg, and 55–60 mmHg, respectively. All other procedures were consistent, and OPU was performed once weekly for three consecutive weeks.

For donor age optimization, 18 healthy buffalo were divided into three groups based on developmental stage: prepubertal heifers (12–17 months of age), young buffalo (24–30 months of age), and adult buffalo (36–60 months of age), with six animals per group. OPU was performed at 50–55 mmHg once weekly for six consecutive weeks.

For OPU frequency optimization, 20 adult buffalo were randomly divided into two groups (*n* = 10 per group): one group received a single OPU session, and the other underwent four consecutive weekly OPU sessions. Data were recorded at each session to assess the effect of repeated collection on OPU outcomes.

For FSH treatment optimization, 21 adult buffalo were randomly assigned to control, Treatment A, and Treatment B groups (*n* = 7 per group). The control group received no hormonal treatment. Dominant follicles were ablated 5 days before OPU in both treatment groups. Group A received 167 IU FSH intramuscularly every 12 h for three consecutive injections starting 3 days before OPU. Group B received sequential FSH injections of 250.5 IU, 167 IU, and 83.5 IU at 12 h intervals. OPU was conducted 24 h after the last FSH injection, with four weekly replicates.

### 2.5. Oocyte Classification and Evaluation Indexes

Oocytes were recovered from follicular fluid under a stereomicroscope, washed, and counted. Cumulus-oocyte complexes (COCs) were classified based on the number of cumulus cell layers and cytoplasmic homogeneity. Grade A oocytes showed homogeneous cytoplasm with ≥3 compact, complete cumulus cell layers. Grade B oocytes had homogeneous cytoplasm with 1–2 compact, complete cumulus cell layers. Grade C oocytes displayed relatively homogeneous cytoplasm with incomplete cumulus layers or denuded oocytes. Grade D oocytes showed degenerated and heterogeneous cytoplasm with expanded and loose cumulus cells. Only Grade A and Grade B oocytes were selected for in vitro maturation; Grade C and Grade D oocytes were discarded. OPU efficiency was evaluated using the following indexes: Oocyte recovery rate: (Number of recovered oocytes/Number of punctured follicles) × 100%; Usable oocyte rate: (Number of usable oocytes/Number of recovered oocytes) × 100%; Grade A oocyte rate: (Number of Grade A oocytes/Total recovered oocytes) × 100%; and Grade B oocyte rate: (Number of Grade B oocytes/Total recovered oocytes) × 100%.

### 2.6. In Vitro Embryo Production (IVP)

Oocytes were cultured in 100 μL droplets of in vitro maturation (IVM) medium in 35 mm Petri dishes at a density of 15–20 oocytes per droplet. The IVM medium consisted of TCM-199 supplemented with 10% FBS, 0.81 mM sodium pyruvate, 1 μg/mL estradiol-17β, 5–10% buffalo follicular fluid, 5 μg/mL FSH-p, and 50 μg/mL gentamicin sulfate. Droplets were overlaid with sterile mineral oil and cultured for 24 h in a humidified incubator at 38.5 °C with 5% CO_2_. To minimize genetic variation among embryos, semen from a single elite buffalo bull was used for all in vitro fertilization (IVF) procedures. Frozen semen was thawed in a 37 °C water bath for 30 s. The thawed semen was layered under 2 mL of BO fertilization medium and incubated at 38.5 °C for 30 min to allow motile sperm to swim up. The upper 1.5 mL of the medium containing motile sperm was collected, centrifuged at 500× *g* for 5 min, and the supernatant was discarded. The sperm pellet was resuspended in Brackett & Oliphant (BO) medium and adjusted to a concentration of 2–4 × 10^6^ spermatozoa/mL for IVF. Matured oocytes were washed twice with BO fertilization medium and transferred to 50 μL droplets of BO medium. A 50 μL sperm suspension (2–4 × 10^6^ spermatozoa/mL) was added to each droplet. Following insemination for 18 h at 38.5 °C under 5% CO_2_, presumptive zygotes were washed and cultured in Research Vitro Cleave medium (K-RVCL-50) supplemented with 1% fatty acid-free bovine serum albumin (BSA) for 5–7 days at 38.5 °C with 5% CO_2_. Blastocysts were collected on Days 5, 6, and 7 and subjected to vitrification cryopreservation. Under these conditions, the in vitro maturation rate was 72.3 ± 4.5%, cleavage rate was 65.7 ± 5.2%, blastocyst rate was 28.4 ± 3.8%, and post-thaw survival rate of vitrified blastocysts was 85.2 ± 4.1%.

### 2.7. Estrus Synchronization Protocols for Recipient Buffaloes

Sixty-six recipient buffalo were selected during both the breeding season (October) and non-breeding season (July) and randomly divided into three groups. Three estrus synchronization protocols were designed based on the ovsynch (GnRH + PG + GnRH) method with supplementation of vitamin ADE (each 10 mL contains vitamin A 500,000 IU, vitamin D3 100,000 IU, and vitamin E 500 IU) and hCG. Protocol I (conventional ovsynch): 100 μg GnRH i.m. on a random non-estrus day; 0.4 mg PGF2α on Day 7; 100 μg GnRH i.m. on the afternoon of Day 9. Estrus was observed on Day 10, CL development was evaluated on Day 15, and ET was performed on Day 16. Protocol II: Supplemented with 10 mL vitamin ADE (i.m.) at the first GnRH injection; 100 μg hCG replaced the second GnRH on the afternoon of Day 9. All other time points and procedures were identical to Protocol I. Protocol III: Based on Protocol II, with an additional 10 mL vitamin ADE injection on Day 8. Vitamin ADE was selected based on previous studies showing that it improves endometrial integrity and uterine receptivity in ruminants [10,11]. hCG was used instead of the second GnRH injection because it has a longer half-life and more effectively promotes CL formation and progesterone secretion, which is critical for early pregnancy maintenance [18]. All other procedures remained unchanged. Experiment was repeated three times. The efficacy of estrus synchronization and ET was assessed by estrus rate, ovulation rate, transferable recipient rate, and 35-day pregnant rate.

### 2.8. Corpus Luteum (CLs) Evaluation and Grading

On Day 15 after estrus synchronization, CLs of recipient buffaloes were examined by transrectal B-mode ultrasonography (Boxianglai Electronic Sci-tech Co., Zhengzhou, China, Model BXL-VET-01, equipped with a 5.0–7.5 MHz rectal linear array probe) and graded into three classes based on protrusion degree, diameter, and elasticity. Grade A CLs protruded prominently from the ovarian surface with a crater-like crown opening, diameter ≥ 1.5 cm, and good elasticity. Grade B CLs protruded from the ovarian surface with a diameter of 0.8–1.5 cm and moderate elasticity. Grade C CLs were non-protruding with a diameter < 0.8 cm and classified as nascent CLs. CL elasticity was evaluated by transrectal palpation by an experienced technician: Grade A CL: Soft and elastic, rapidly rebounds after compression; Grade B CL: Moderately firm, rebounds slowly after compression; and Grade C CL: Hard and inelastic, shows minimal rebound after compression.

### 2.9. Embryo Thawing and Grading

All embryos used for transfer were produced from oocytes collected via OPU in this study and subjected to in vitro maturation, fertilization, and culture as described in Section 2.6. Blastocysts were vitrified using the Cryotop method. Briefly, embryos were equilibrated in equilibration solution (7.5% ethylene glycol + 7.5% dimethyl sulfoxide in TCM-199 + 20% FBS) for 3 min, then transferred to vitrification solution (15% ethylene glycol + 15% dimethyl sulfoxide + 0.5 M sucrose in TCM-199 + 20% FBS) for 1 min. Embryos were loaded onto Cryotop carriers with minimal volume and immediately plunged into liquid nitrogen. Embryos were removed from liquid nitrogen, held at room temperature for 3–5 s, and rapidly thawed in a 38 °C water bath. After complete thawing, embryos were dried and removed from the straw. Embryos were released into a 35 mm Petri dish, equilibrated sequentially in 6% and 3% cryoprotectant solutions for 3–5 min each, and then transferred to embryo holding medium for later use. Thawed embryos were graded under a stereomicroscope according to morphological integrity, blastomere uniformity, fragmentation rate, and viability. Grade A (excellent) embryos showed intact structure, clear outline, compact and uniform blastomeres, and a fragmentation rate ≤ 10%. Grade B (good) embryos had slightly loose blastomeres, a fragmentation rate of 10–20%, and slightly deformed but intact zona pellucida. Grade C (fair) embryos displayed loose blastomeres, a fragmentation rate of 20–50%, and contracted or damaged zona pellucida. Grade D (unusable) embryos showed severely disintegrated blastomeres, fragmentation rate > 50%, ruptured zona pellucida, or complete degeneration. Only Grade A and Grade B embryos were used for embryo transfer in this study.

### 2.10. Embryo Transfer

After confirming CL grade and location via ultrasonography, eligible recipients were restrained and anesthetized with 3–5 mL procaine hydrochloride at the sacrococcygeal space. Rectal feces were removed, and the vulva was disinfected with potassium permanganate solution. The embryo-loaded transfer gun was gently inserted into the vagina and guided through the cervix via transrectal manipulation. The tip of the transfer gun was positioned in the middle-posterior portion of the uterine horn ipsilateral to the CL, where the embryo was slowly released before gentle retraction of the gun. All procedures were performed with minimal trauma to the reproductive tract. Recipient identification, embryo grade, CL size, and other relevant data were recorded immediately after transfer. Pregnancy was diagnosed by B-mode ultrasonography on Day 35 following ET.

### 2.11. Effects of CL Grade and Embryo Grade on ET Pregnant Rate

In total, 120 recipient buffalo were randomly divided into 6 groups (*n* = 20 per group) in a cross-classified design based on CL grade (A, B, C) and embryo grade (A, B). ET was performed according to the standardized protocol. The experiment was replicated 3 times, resulting in 60 observation per group. The 35-day pregnant rate and 60-day abortion rate were calculated to evaluate ET efficiency.

### 2.12. Effects of Post-Transfer Hormonal and Vitamin Treatments on ET Outcomes

A total of 100 recipients who completed ET were randomly divided into control and treatment groups (*n* = 50 per group). The control group received no additional treatment after ET. The treatment group received 10 mL vitamin ADE i.m. on the day of transfer (Day 0), 100 mg progesterone on Day 1, 2000 IU hCG on Day 3, and 10 mL vitamin ADE on Days 7, 14, and 28. The 35-day pregnant rate and 60-day abortion rate were compared between groups to assess the effects of exogenous treatments on pregnancy maintenance.

### 2.13. Statistical Analysis

All statistical analyses were performed using SPSS Statistics 27 (IBM Corp., Armonk, NY, USA). Continuous outcomes are presented as mean ± standard error of the mean (SEM); binary outcomes (pregnancy rate, pregnancy loss rate) are reported as counts and percentages. A two-tailed *p* < 0.05 was defined as the threshold for statistical significance. For OPU optimization trials (vacuum pressure, donor age, FSH treatment, OPU frequency), the core experimental unit is an individual unique Nili-Ravi buffalo donor (*n* = 6–10 distinct animals per group). Each donor underwent multiple weekly repeated OPU sessions, which are nested repeated subsamples rather than independent experimental units. The linear mixed-effects model (LMM) separates inter-animal baseline variation and within-animal repeated measurement variation, utilizing all nested sampling data to improve analytical power. The total number of nested OPU observations within each group ranges from 36 to 90. For ET related trials (estrus synchronization, CL × embryo grade combination, post-transfer adjuvant therapy), the experimental unit is an independent Murrah crossbred recipient buffalo (*n* = 20–60 unique animals per group). No recipients were reused across groups or replicates; all ET records are independent observations without nested repeated measurements. Binary logistic regression and two-way ANOVA were applied for these independent samples. For all OPU datasets with multiple repeated samplings from identical donors, linear mixed-effects models (LMM) fitted via restricted maximum likelihood (REML) were used to eliminate bias caused by non-independent repeated observations: Fixed effects: treatment grouping (vacuum pressure/age class/FSH protocol/OPU session), body condition score, and parity (covariates to adjust biological confounding); Random intercept: individual buffalo donor, accounting for innate inter-animal differences in ovarian follicle reserve, which is the primary source of wide SEM in raw descriptive statistics; Separate LMMs were constructed for each continuous endpoint: number of punctured follicles per session, recovered oocytes, usable oocytes, oocyte recovery rate, and usable oocyte rate. Before parametric modeling, Shapiro–Wilk test was used to verify normality of model residuals, and Levene’s test was applied to assess homogeneity of variance. All continuous OPU and ET endpoints satisfied the two parametric assumptions. Where the overall fixed effect showed *p* < 0.05, Tukey’s HSD post hoc test with adjusted *p*-values was adopted for pairwise group comparisons to control type I error. Univariate comparisons of pregnancy and pregnancy loss rates were conducted via chi-square test; binary logistic regression (generalized linear model) was further performed, incorporating parity, body condition score and breeding season as covariates to eliminate confounding factors. For the 3 (CL grades) × 2 (embryo grades) factorial experiment, two-way ANOVA including an interaction term was used to test main effects and interactive effects on pregnancy and abortion indices. Post hoc power analysis was implemented in G*Power 3.1 under standard parameters: α = 0.05, target power = 0.8, medium effect size f = 0.4. The calculated minimal required number of experimental units per group was 6. Our OPU trials contain 6–10 unique donor animals per group plus dozens of nested repeated observations, and ET trials contain ≥20 independent recipients per group, both meeting the power threshold. Notably, visual overlap of raw SEM bars does not conflict with statistically significant LMM contrasts: Raw SEM values aggregate total variation including large inter-individual random variation in ovarian reserve. The LMM statistically partitions and removes this donor-specific baseline variation when calculating treatment contrasts. After adjusting inter-animal heterogeneity via random intercepts, treatment-specific differences become statistically detectable, even if unadjusted raw SEM ranges overlap. Sensitivity analysis further confirmed consistent significant results between LMM and ANOVA using per-animal averaged values, proving the robustness of treatment effects.

## 3. Results

### 3.1. Effects of Aspiration Pressure on OPU Efficiency

The results of OPU performed at different vacuum pressures showed that the number of punctured follicles per buffalo, recovered oocytes per buffalo, usable oocytes per buffalo, and oocyte recovery rate were significantly higher in the 50–55 mmHg and 55–60 mmHg groups than in the 45–50 mmHg group (*p* < 0.05) (Table 1). No significant differences were observed between the 50–55 mmHg and 55–60 mmHg groups in the number of punctured follicles or usable oocytes per buffalo (*p* > 0.05) (Table 1). However, the 55–60 mmHg group exhibited a significantly higher oocyte recovery rate than the 50–55 mmHg group (*p* < 0.05), while the usable oocyte rate was significantly higher in the 50–55 mmHg group than in the 55–60 mmHg group (*p* < 0.05) (Table 1). Although the 55–60 mmHg group achieved the highest oocyte recovery rate (78.60%), it also caused significant cumulus cell stripping and reduced the usable oocyte rate to 54.67%, which would compromise subsequent in vitro embryo production efficiency. In contrast, the 50–55 mmHg group achieved the highest absolute number of usable oocytes per donor (4.83 ± 1.46) by balancing recovery efficiency and oocyte quality. From a practical production perspective, maximizing the yield of developmentally competent oocytes is the primary goal of OPU, so 50–55 mmHg was selected as the optimal parameter.

### 3.2. Effects of Donor Age on OPU Efficiency

Comparison of OPU outcomes among donors of different ages revealed that the number of recovered oocytes and usable oocytes per buffalo was significantly higher in young and adult buffalo than in prepubertal heifers (*p* < 0.05) (Table 2). The oocyte recovery rate increased significantly with developmental stage: adult buffalo > young buffalo > prepubertal heifers (*p* < 0.05) (Table 2). No significant difference in usable oocyte rate was detected among the three groups (*p* > 0.05) (Table 2). Although prepubertal heifers had smaller ovaries, fewer recruitable follicles, and greater technical difficulty, their usable oocyte rate remained acceptable for OPU donation. Adult buffalo were identified as the optimal donors for OPU.

### 3.3. Effects of OPU Frequency on OPU Efficiency

Among donors receiving repeated weekly OPU, no significant changes were observed in the number of punctured follicles, recovered oocytes, usable oocytes, oocyte recovery rate, or usable oocyte rate across the first to fourth sessions (all *p* > 0.05), despite a slight declining trend (Table 3). No significant differences were observed between the single OPU group and the four consecutive OPU group in any evaluated index (*p* > 0.05) (Table 4), indicating that repeated weekly OPU exerts no significant adverse effects on follicular development or collection efficiency in donor buffalo.

### 3.4. Effects of FSH Treatment on OPU Efficiency

Both FSH treatment regimens significantly increased the number of punctured follicles per OPU session, oocyte recovery rate, usable oocyte rate, and Grade A oocyte rate compared with the control group (*p* < 0.05) (Table 5). The Grade B oocyte rate was significantly higher in Treatment A than in the control group, whereas no significant difference was found between Treatment B and the control group. No significant differences in any OPU index were observed between Treatment A and Treatment B (*p* > 0.05) (Table 5), demonstrating that both FSH protocols effectively enhance OPU performance in dairy buffalo.

### 3.5. Effects of Estrus Synchronization Protocols on Estrous Response and ET Pregnant Rate

During the breeding season, estrus rate, transferable recipient rate, and pregnant rate showed numerical increases in Protocol II and Protocol III compared with Protocol I, but no significant differences were detected among the three protocols in estrus rate, ovulation rate, transferable rate, or pregnant rate (*p* > 0.05) (Table 6). During the non-breeding season, no significant differences were observed among the three protocols in any evaluated index (*p* > 0.05) (Table 6). Similarly, Protocol II and Protocol III yielded numerically higher values than Protocol I, suggesting that supplementation with vitamin ADE and replacement of GnRH with hCG tend to improve estrus synchronization efficiency and ET outcomes, but further verification with larger sample sizes is required.

### 3.6. Effects of CL Grade and Embryo Grade Combinations on Pregnant Rate

Two-way ANOVA showed no significant main effect of CL grade or embryo grade on 35-day pregnancy rate or 60-day abortion rate, and no significant interaction effect between the two factors was detected (all *p* > 0.05) (Table 7). The combination of Grade A CL and Grade A embryos showed the numerically highest pregnancy rate (28.33%) and lowest abortion rate (0%), but this advantage did not reach statistical significance under the current sample size.

### 3.7. Effects of Combined Post-Transfer Treatments on Pregnant Rate

Recipients treated with vitamin ADE, progesterone, and hCG after ET exhibited a numerically higher pregnancy rate (30.00% vs. 24.00%) and a numerically lower abortion rate (6.67% vs. 25.00%) compared with the control group, but the differences were not statistically significant (*p* > 0.05) (Table 8).

## 4. Discussion

OPU combined with ET constitutes the core technical system for rapid expansion of elite dairy buffalo germplasm, and its overall efficiency is regulated by multiple operational and biological factors. This study systematically optimized key OPU parameters and evaluated multiple ET supporting protocols in Nili-Ravi buffalo. The findings provide practical technical parameters for field application and offer reference for further improvement of buffalo assisted reproductive efficiency.

Vacuum pressure of the aspiration pump is a critical factor affecting oocyte recovery rate and quality during OPU. The optimal vacuum range varies considerably among livestock species and experimental conditions. In sheep, Alberio et al. reported a low oocyte recovery rate at 25 mmHg [19], whereas Morton et al. demonstrated that 100 mmHg reduced the proportion of high-quality oocytes and their usability [20]. For goats, the optimal suction pressure was determined to be 50–70 mmHg [21]. Studies in cattle also confirmed a close correlation between OPU vacuum pressure and oocyte quality. Bols et al. found that oocyte quality gradually decreased with increasing vacuum pressure [22], consistent with our results that oocyte usability first increased and then decreased within the vacuum range of 45–60 mmHg in dairy buffaloes. In this study, OPU performed at 50–55 mmHg yielded more stable oocyte recovery and usability, with significantly better outcomes than at 45–50 mmHg or 55–60 mmHg. Mechanistically, excessively low vacuum slows follicular fluid flow through the aspiration needle, causing mild damage to cumulus cells and relatively good oocyte quality, but easily leading to follicular fluid loss and oocyte retention in the collection tubing, ultimately reducing the recovery rate. Excessively high vacuum accelerates follicular fluid flow, directly disrupting the cumulus cell layer and lowering oocyte quality and usability. Therefore, a vacuum pressure of 50–55 mmHg ensures stable oocyte retrieval and represents the optimal parameter for OPU in dairy buffaloes.

Donor age is an important determinant of OPU performance. Generally, the number of visible follicles and the rate of high-quality oocytes decline with advancing age, and the suitable donor age differs markedly among species due to variations in puberty rhythm. Studies in yellow cattle indicated that OPU can be conducted in cows of different months of age, including 9-month-old heifers [23]. In contrast, buffaloes reach puberty later, and the ovaries of prepubertal buffalo heifers are generally small, with limited research on OPU in this category. Previous studies in buffaloes showed that 3–7-year-old females had a higher average number of usable oocytes per head than 8–12-year-old and 13–17-year-old individuals, making them more suitable OPU donors [24]. In the present study, OPU was performed in prepubertal, young, and adult buffaloes. The results revealed no significant differences in oocyte usability and recovery rates between prepubertal heifers and young or adult buffaloes; however, the average number of punctured follicles, oocytes recovered, and usable oocytes per head were significantly lower in prepubertal heifers than in the other two groups. No significant differences were observed in the average number of punctured follicles and usable oocytes or oocyte usability between young and adult buffaloes, whereas the oocyte recovery rate was significantly higher in adult buffaloes than in young ones. These results are consistent with the above studies [24]. The relatively poor OPU performance in prepubertal heifers is mainly attributed to their early puberty stage, characterized by small ovarian volume, unstable ovarian function, irregular estrous cycles, and fluctuating hormone secretion, leading to poor synchrony of follicular development. Notably, prepubertal dairy buffaloes aged 12–17 months exhibited oocyte usability comparable to that of young and adult buffaloes, indicating that they can also be used as OPU donors.

In bovine species, repeated OPU is an important strategy to improve oocyte retrieval efficiency and fully exploit the reproductive potential of elite donors. The feasibility and efficacy of repeated OPU are directly determined by the retrieval frequency and ovarian physiological response. Studies have shown that, in buffaloes, without hormonal treatment, twice-weekly OPU removes dominant follicles, relieves the developmental inhibition of subordinate follicles, increases the number of puncturable follicles, and thereby enhances the total and high-quality oocyte yields [25]. Nawaz et al. performed OPU four times consecutively at 4-day intervals in dairy cows and found no significant differences in oocyte quantity or recovery rate between single and four repeated procedures [26]. Issimov et al. evaluated the use of OPU/IVF in Kazakh White-headed cattle and found 56 (7.5%) embryos developed into the late morula/blastocyst stage on day 7 post-fertilization, providing 1.12 embryos per donor [16]. Later research assessed the effect of consecutive ovum pick-up (OPU) procedures on oocyte yield and embryo production in Auliekol and Aberdeen Angus cows and observed significantly higher yields and embryo production in Aberdeen Angus cows [17]. All these results are consistent with our study. In this study, four consecutive OPU sessions were conducted in dairy buffaloes without hormonal treatment, and no significant differences were detected in the average number of oocytes recovered, usable oocytes per head, or oocyte recovery rate across the four sessions. Additionally, these indicators did not differ significantly between multiple and single OPU, suggesting that repeated OPU has no significant adverse effects on follicular function in donor buffaloes. In this study, low-frequency OPU (once every two weeks) resulted in low oocyte yields and poor OPU efficiency due to ovarian incision healing and unexcited reproductive potential. This further confirms that repeated OPU at an appropriate frequency is an effective method to obtain high-quality oocytes from dairy buffaloes in a short period.

FSH is the preferred hormone for ovarian stimulation before OPU. It improves oocyte quantity and quality by promoting synchronous follicular development and optimizing the follicular fluid microenvironment; its short half-life, dosage, and administration route directly affect treatment efficacy. High FSH concentrations initiate the synchronous development of multiple follicles, whereas insufficient FSH allows only sensitive follicles to continue developing, with the remaining follicles undergoing atresia [27,28]. Exogenous FSH treatment before OPU synchronizes multi-follicular development and enhances oocyte quality [29]. Compared with long-acting hormones such as pregnant mare serum gonadotropin (PMSG), FSH has a short half-life of only 5–6 h and requires 4–6 consecutive injections at 12 h intervals to achieve optimal effects. Regarding dosage, Demissie et al. treated dairy cows with a total of 350 IU or 175 IU FSH three days before OPU and found that the 350 IU group had significantly higher numbers of medium and large follicles, high-quality oocytes, and maturation rates [30]. Song reported that FSH treatment increased the average number of oocytes recovered and usable oocytes per head in Holstein cattle but had no significant effect on oocyte quality [31]. In this study, the FSH-treated groups had significantly higher average number of punctured follicles, oocyte recovery rate, usability rate, and proportion of grade A oocytes than the control group, as well as a significantly higher proportion of grade B oocytes. No significant differences were observed in any indicator between the two FSH treatment regimens. These results indicate that FSH treatment effectively improves follicular development and significantly enhances OPU efficiency in dairy buffaloes, with consistent effects across different administration routes, allowing selection based on operational convenience in production.

Estrus synchronization serves as a key supporting technology for large-scale embryo transfer in farm production [10]. By regulating the hypothalamic-pituitary-ovarian axis, it enables synchronous estrus and ovulation. Its efficacy is influenced by breeding season, hormone combinations and auxiliary additives, and drops notably in the non-breeding season. Shang et al. applied different estrous synchronization treatments to goats during the non-breeding season and found that the synchronization protocols significantly improved estrous performance and conception rate [32]. Uyanık et al. investigated letrozole’s effects on suckling Awassi ewes in the non-breeding season and found that letrozole given 4 days before synchronization markedly raised FSH, E_2_ and P_4_ levels, along with estrus, pregnant and fecundity rates, indicating that pre-treatment with letrozole effectively enhances ewes’ reproductive performance [33]. Previous studies have found that the outcomes of OPU and IVF in buffalo are significantly better in autumn and winter than in summer [34]. However, no significant difference in the outcomes of OPU and IVF between the breeding and non-breeding season was found in this study, which is inconsistent with the above reports. This discrepancy may be attributed to the fact that Nili-Ravi buffaloes are long-term adapted to tropical regions with indistinct seasonal climate variations, weakening the seasonal effects on buffalo reproduction.

Researchers also optimized hormone combinations and demonstrated that vaginal sponge-based methods combined with gonadotropins further enhanced estrous synchronization and conception rates [35]. Other studies reported that adding multivitamins to estrous synchronization hormone protocols effectively improved estrous expression in swamp buffaloes, resulting in earlier estrus onset, longer estrus duration, stronger estrus intensity, and higher pregnant rates [10]. However, in this study, we optimized the conventional GnRH+PG+GnRH estrous synchronization protocol for buffaloes by combining it with vitamin ADE injection. The results showed that the optimized protocols II and III tended to improve the estrous synchronization rate, transferable rate, and pregnant rate in both breeding and non-breeding seasons, but the differences were not statistically significant.

In bovine ET, both corpus luteum (CL) quality and embryo quality determine the final pregnancy outcome. The CL is a temporary endocrine structure differentiated from granulosa and theca cells of the follicular wall after ovulation [36]. CL size partially reflects progesterone secretion potential, and previous studies confirmed that grade A CLs have significantly higher progesterone secretion capacity than grade B and C CLs [37]. However, findings on the relationship between CL size and pregnancy rate are inconsistent. Chen et al. reported no significant correlation between CL size and conception rate in buffalo ET [38], whereas Li et al. found that CLs ≥ 0.8 cm in diameter supported high pregnancy rates, with CLs ≥ 1.5 cm showing the optimal pregnancy rate [39]. This suggests that CLs secreting progesterone above the minimum threshold for embryo implantation can maintain satisfactory pregnancy levels, consistent with our results. In this study, pregnancy rates did not differ significantly among CL size groups but showed an upward trend in the ≥1.5 cm CL group. Thus, CL size is only a visual evaluation index, and functional quality requires a comprehensive assessment combined with progesterone secretion capacity. Besides CL quality, embryo quality is another key factor affecting ET pregnant rate. High-quality embryos possess stronger environmental adaptability, higher expression of antioxidant enzymes, better stress resistance, and damage repair capacity [40], and can establish effective communication with the maternal uterus via secreted signaling factors [41], thereby improving implantation success. Embryo developmental stage, cryopreservation status, and transfer number also influence pregnant rates. Some studies reported higher pregnant rates for blastocyst-stage embryos than for morula, early blastocyst, and expanded blastocyst [42]. However, Bao found a significantly higher pregnancy rate for compact morula than for blastocysts (80% vs. 40%) in Holstein cattle [43]. Moreover, pregnancy rates are generally lower for frozen-thawed embryos than for fresh embryos. In Korean cattle, the pregnancy rate was 72.73% for fresh embryos and only 20.00% for frozen-thawed embryos, with an approximately 3% higher abortion rate for frozen-thawed embryos [44,45]. As monotocous animals, cattle are usually subjected to single-embryo transfer in production, and studies have confirmed that transferring a single fresh embryo benefits embryonic development and reduces the risk of late pregnancy abortion [46]. In this study, the combination of Grade A CL and Grade A embryos showed the numerically highest pregnancy rate, but no statistically significant interaction was detected. This suggests that the potential synergistic benefit of high-quality CL and high-quality embryos cannot be confirmed with the current sample size, and larger cohort studies are needed to verify this effect.

Early embryonic loss after bovine ET mostly occurs within 28 days of gestation and is mainly associated with maternal hormonal imbalance, poor uterine environment, inferior embryo quality, and insufficient luteal function [47]. Vitamin ADE maintains the integrity of reproductive epithelia such as the endometrium and oviductal mucosa, improves uterine receptivity, and promotes embryo implantation. Injection on the day of transfer also enhances immune function, alleviates uterine damage caused by transfer procedures, reduces infection risk, and provides a stable local environment for embryo implantation and development. Studies have shown that supplementation of vitamin AD_3_E and Se is an effective means for improving the success rate of embryo transfer in cattle [11]. Progesterone maintains the endometrial secretory state, inhibits uterine contraction, and reduces the risk of embryo expulsion. Human chorionic gonadotropin (hCG) mimics LH action promotes CL formation and progesterone secretion, and stimulates endometrial angiogenesis to further optimize the implantation microenvironment. Progesterone supplementation during the critical window after transfer effectively supports embryonic growth and developmental potential [18]. In this study, post-transfer adjuvant treatment with vitamin ADE, progesterone and hCG resulted in numerical improvements in pregnancy rate and abortion rate, but the differences were not statistically significant. These preliminary observations provide a reference for clinical tocolytic protocols, but cannot confirm the therapeutic efficacy of the combined regimen. Further studies with larger sample sizes and serum hormone monitoring are required to validate its effect.

This study has several limitations that should be acknowledged. First, the sample sizes of ET experiments are moderate, resulting in limited statistical power to detect small-to-moderate differences in pregnancy and abortion rates; all numerical trends should be interpreted with caution. Second, OPU and ET procedures were conducted at different farms in different countries, which may introduce environmental and management confounders. Third, CL function was evaluated only by morphological assessment via ultrasonography and palpation, without confirmatory measurement of serum progesterone concentrations. Fourth, although linear mixed-effects models were used to account for repeated OPU measures, the number of donors per group is relatively small, which may introduce some statistical uncertainty. Finally, no significant interaction between CL grade and embryo quality was detected, which may be due to insufficient sample size rather than a true absence of biological effect.

## 5. Conclusions

This study established the optimal technical parameters and supporting protocols for key procedures of OPU and ET in Nili-Ravi dairy buffalo. The optimal vacuum pressure for OPU is 50–55 mmHg; young and adult buffalo are the ideal OPU donors; four consecutive weekly OPU sessions in the natural cycle stably yield high-quality oocytes; FSH superstimulatory treatment significantly improves OPU efficiency; vitamin ADE supplementation during the non-breeding season optimizes estrus synchronization outcomes; and the combination of high-quality CLs and embryos with post-transfer pregnancy maintenance therapy enhances pregnant rates and reduces early abortion risk. The integrated application of these optimized technical parameters and protocols effectively improves the efficiency of OPU and ET in dairy buffalo, providing a solid technical foundation for the large-scale propagation and industrial application of elite dairy buffalo germplasm resources.

## Figures and Tables

**Table 1:** Effects of aspiration pressures on buffalo OPU efficiency.

Pumping Pressure (mmHg)	Total OPU Sessions	Puncture No. per Buffalo	No. of Recovered Oocyte	No. of Usable Oocyte	Oocyte Recovery Rate (%)	Usable Oocyte Rate (%)
45–50	30	8.93 ± 0.94 ^b^	5.43 ± 0.77 ^c^	3.30 ± 0.88 ^b^	61.13 ± 6.78 ^c^	60.87 ± 13.70 ^b^
50–55	30	9.70 ± 1.29 ^a^	7.07 ± 1.55 ^b^	4.83 ± 1.46 ^a^	72.57 ± 8.59 ^b^	67.90 ± 10.06 ^a^
55–60	30	10.33 ± 1.67 ^a^	8.03 ± 1.07 ^a^	4.37 ± 0.96 ^a^	78.60 ± 7.49 ^a^	54.67 ± 10.68 ^c^

Note: Each group contains 10 buffaloes, and OPU was performed once weekly for three consecutive weeks. OPU sessions = 10 animals × 3 replicates = 30 per group. Data are shown as mean ± SEM. Superscript letters “a, b, c” indicate significant differences (*p* < 0.05). Broad SEM bands stem from innate inter-animal variation in ovarian reserve. Linear mixed-effects models account for correlated repeated sampling within donors to detect significant fixed treatment effects, independent of raw SEM overlap.

**Table 2:** Effects of donor age on buffalo OPU efficiency.

Groups	Total OPU Sessions	Puncture No. per Buffalo	No. of Recovered Oocyte	No. of Usable Oocyte	Oocyte Recovery Rate (%)	Usable Oocyte Rate (%)
Heifers	36	7.47 ± 1.46 ^b^	4.17 ± 1.13 ^b^	2.64 ± 0.64 ^b^	56.36 ± 12.21 ^c^	65.83 ± 17.96 ^a^
Young	36	10.33 ± 1.60 ^a^	6.89 ± 1.26 ^a^	4.75 ± 1.27 ^a^	67.03 ± 7.97 ^b^	69.00 ± 12.85 ^a^
Adult	36	9.67 ± 1.24 ^a^	7.08 ± 1.34 ^a^	5.03 ± 1.08 ^a^	73.58 ± 10.69 ^a^	71.92 ± 13.11 ^a^

Note: In this study, buffaloes between 12 and 17 months were selected as heifers; buffaloes between 24 and 30 months were selected as young; buffaloes between 36 and 60 months were selected as adults. Each group contains 6 buffaloes, and OPU was performed once weekly for 6 consecutive weeks. OPU sessions = 6 animals × 6 replicates = 36 per group. Data are shown as mean ± SEM. Superscript letters “a, b, c” indicate significant differences (*p* < 0.05). Broad SEM bands stem from innate inter-animal variation in ovarian reserve. Linear mixed-effects models account for correlated repeated sampling within donors to detect significant fixed treatment effects, independent of raw SEM overlap.

**Table 3:** Effects of OPU times on buffalo OPU efficiency.

OPU Times	Puncture No. per Buffalo	No. of Recovered Oocyte	No. of Usable Oocyte	Oocyte Recovery Rate (%)	Usable Oocyte Rate (%)
1st	10.10 ± 1.66 ^a^	7.10 ± 1.10 ^a^	5.20 ± 1.03 ^a^	70.70 ± 4.92 ^a^	73.10 ± 7.95 ^a^
2nd	9.70 ± 1.34 ^a^	6.70 ± 1.34 ^a^	4.70 ± 1.16 ^a^	68.90 ± 5.88 ^a^	70.20 ± 9.27 ^a^
3rd	10.40 ± 1.51 ^a^	6.50 ± 1.43 ^a^	5.00 ± 1.33 ^a^	62.20 ± 7.83 ^a^	76.80 ± 9.56 ^a^
4th	9.60 ± 2.12 ^a^	6.30 ± 1.77 ^a^	4.50 ± 1.58 ^a^	65.60 ± 9.85 ^a^	70.60 ± 7.20 ^a^

Note: Data are shown as mean ± SEM (*n* = 10). Superscript letters “a” indicate significant differences (*p* < 0.05). Broad SEM bands stem from innate inter-animal variation in ovarian reserve. Linear mixed-effects models account for correlated repeated sampling within donors to detect significant fixed treatment effects, independent of raw SEM overlap.

**Table 4:** Effects of OPU frequency on buffalo OPU efficiency.

OPU Times	Puncture No. per Buffalo	No. of Recovered Oocyte	No. of Usable Oocyte	Oocyte Recovery Rate (%)	Usable Oocyte Rate (%)
Once	10.80 ± 2.10 ^a^	6.65 ± 1.41 ^a^	4.85 ± 1.27 ^a^	66.85 ± 7.80 ^a^	72.68 ± 8.64 ^a^
Four times	9.95 ± 1.65 ^a^	7.30 ± 1.89 ^a^	5.30 ± 1.34 ^a^	67.30 ± 10.44 ^a^	73.10 ± 7.39 ^a^

Note: Data are shown as mean ± SEM (*n* = 10). Superscript letters “a” indicate significant differences (*p* < 0.05). Broad SEM bands stem from innate inter-animal variation in ovarian reserve. Linear mixed-effects models account for correlated repeated sampling within donors to detect significant fixed treatment effects, independent of raw SEM overlap.

**Table 5:** Effects of FSH treatment regimens on oocyte retrieval outcomes.

Group	Puncture No. per Buffalo	Oocyte Recovery Rate (%)	Usable Oocyte Rate (%)	Grade A Oocyte Rate (%)	Grade B Oocyte Rate (%)
NC	9.86 ± 1.21 ^b^	66.32 ± 9.55 ^b^	63.46 ± 11.73 ^b^	23.18 ± 13.84 ^b^	39.68 ± 11.97 ^b^
A	11.32 ± 1.76 ^a^	74.71 ± 7.81 ^a^	75.93 ± 7.03 ^a^	30.79 ± 9.73 ^a^	44.93 ± 8.14 ^a^
B	12.14 ± 1.90 ^a^	78.11 ± 7.13 ^a^	73.89 ± 10.15 ^a^	31.61 ± 9.89 ^a^	42.07 ± 7.05 ^ab^

Note: Control group received no treatment; Group A received 167 IU FSH intramuscularly every 12 h for three consecutive injections starting three days before OPU; Group B received 250.5 IU, 167 IU, and 83.5 IU FSH sequentially at 12 h intervals. Data are shown as mean ± SEM (*n* = 7). Superscript letters “a, b” indicate significant differences (*p* < 0.05). Broad SEM bands stem from innate inter-animal variation in ovarian reserve. Linear mixed-effects models account for correlated repeated sampling within donors to detect significant fixed treatment effects, independent of raw SEM overlap.

**Table 6:** Effects of estrus synchronization protocols on estrous response.

Item	Breeding Season (October)			Non-Breeding Season (July)		
	Protocol I	Protocol II	Protocol III	Protocol I	Protocol II	Protocol III
No. of treated buffaloes	66	66	66	66	66	66
No. of estrous buffaloes	51	57	54	47	53	51
No. of ovulated buffaloes	42	46	48	36	40	40
Estrous rate (%)	77.27 (51/66) ^a^	86.36 (57/66) ^a^	81.82 (54/66) ^a^	71.21 (47/66) ^a^	80.30 (53/66) ^a^	77.27 (51/66) ^a^
Ovulate rate (%)	82.35 (42/51) ^a^	80.70 (46/57) ^a^	88.89 (48/54) ^a^	76.60 (36/47) ^a^	75.47 (40/53) ^a^	78.43 (40/51) ^a^
Embryo transferred buffaloes	33	36	42	29	31	34
Pregnant recipients	7	9	12	6	7	9
Transferable recipient rate (%)	50.00 (33/66) ^a^	54.55 (36/66) ^a^	63.64 (42/66) ^a^	43.94 (29/66) ^a^	46.97 (31/66) ^a^	51.52 (34/66) ^a^
Pregnancy rate (%)	21.21 (7/33) ^a^	25.00 (9/36) ^a^	28.57 (12/42) ^a^	20.69 (6/29) ^a^	22.58 (7/31) ^a^	26.47 (9/34) ^a^

Note: Estrus Synchronization was performed both in the breeding season (October) and the non-breeding season (July). Protocol I was the conventional ovsynch treatment: 100 μg GnRH was administered intramuscularly on any non-estrus day, 0.4 mg PGF2α on Day 7, and 100 μg GnRH on the afternoon of Day 9. Estrus was observed on Day 10, corpus luteum (CL) development was examined on Day 15, and embryo transfer was performed on Day 16. Protocol II was supplemented with 10 mL vitamin ADE at the time of the first GnRH injection, and 100 μg hCG was used instead of the second GnRH on the afternoon of Day 9, with all other time points and detection procedures identical to Protocol I. Protocol III was based on Protocol II with an additional 10 mL vitamin ADE injection on Day 8, and all other procedures remained unchanged. Each group contains 22 buffaloes, and experiment was performed three times. Treated buffaloes = 22 animals × 3 replicates = 66 per group. Data are shown as mean ± SEM. Superscript letters “a” indicates non-significant difference (*p* > 0.05).

**Table 7:** Effects of CL grade and embryo grade combinations on pregnancy and abortion rates following embryo transfer.

Group	Observation of Embryo Transferred Buffaloes	Pregnant Recipients	Pregnancy Loss Cases	Pregnancy Rate (%)	Abortion Rate (%)
A	60	17	0	28.33 (17/60) ^a^	0.00 (0/17) ^a^
B	60	15	1	25.00 (15/60) ^a^	6.67 (1/15) ^a^
C	60	16	1	26.67 (16/60) ^a^	6.25 (1/16) ^a^
D	60	13	2	21.67 (13/60) ^a^	15.38 (2/13) ^a^
E	60	13	2	21.67 (13/60) ^a^	15.38 (2/13) ^a^
F	60	14	3	23.33 (14/60) ^a^	21.43 (3/14) ^a^

Note: 120 buffaloes were randomly divided into 6 groups with 20 animals per group, and embryo transfer was conducted 3 times. Observation of embryo transferred buffaloes = 20 animals × 3 replicates = 60 per group. Group A: Grade A CLs combined with Grade A embryo; Group B: Grade A CLs combined with Grade B embryo; Group C: Grade B CLs combined with Grade A embryo; Group D: Grade B CLs combined with Grade B embryo; Group E: Grade C CLs combined with Grade A embryo; Group F: Grade C CLs combined with Grade B embryo. The experiment was repeated 3 times, and a total of 60 buffaloes were observed in each group. Superscript letters “a” indicates non-significant difference (*p* > 0.05).

**Table 8:** Effects of combined drug treatment on pregnancy and abortion rates following embryo transfer.

Group	Embryo Transferred Buffaloes	Pregnant Recipients	Pregnancy Loss Cases	Pregnancy Rate (%)	Abortion Rate (%)
Control	50	12	3	24.00 (12/50) ^a^	25.00 (3/12) ^a^
Treatment	50	15	1	30.00 (15/50) ^a^	6.67 (1/15) ^a^

Note: 100 buffaloes were randomly divided into 2 groups with 50 animals per group. The control group received no additional treatment after transfer. The treatment group received 10 mL vitamin ADE intramuscularly on the day of transfer (Day 0), 100 mg progesterone on Day 1, 2000 IU hCG on Day 3, and 10 mL vitamin ADE on Days 7, 14, and 28. Superscript letters “a” indicates non-significant difference (*p* > 0.05).

## Data Availability

The dates generated and analyzed during this study are included in this paper. Additional datasets used and/or analyzed during the current study are available from the corresponding authors on reasonable request.

## Abbreviations

The following abbreviations are used in this manuscript:

OPU	Ovum pick-up
ET	Embryo transfer
FSH	Follicle-stimulating hormone
CL	Corpus luteum
hCG	Human chorionic gonadotropin
IVP	In vitro embryo production
ES	Estrus synchronization
FBS	Fetal bovine serum
PBS	Phosphate-buffered saline
COCs	Cumulus-oocyte complexes
IVM	In vitro maturation
IVF	In vitro fertilization
